# Comparison between minimally invasive deltoid-split and extended deltoid-split approach for proximal humeral fractures: a case-control study

**DOI:** 10.1186/s12891-020-03417-9

**Published:** 2020-06-27

**Authors:** Ji-Qi Wang, Chui-cong Lin, You-Ming Zhao, Bing-Jie Jiang, Xiao-Jing Huang

**Affiliations:** 1 Department of Orthopaedics, The Second Affiliated Hospital and Yuying Children’s Hospital of Wenzhou Medical University, 109# Xue Yuan Xi Road, Wenzhou, 325000 Zhejiang China; 2 Department of Orthopaedics, The First Affiliated Hospital of Wenzhou Medical University, Shangcai Village Ouhai District, Wenzhou, 325000 Zhejiang China

**Keywords:** Proximal humeral fractures, Deltoid-split approach, Minimally invasive approach, Extended approach, Reduction loss

## Abstract

**Background:**

With the rapid aging of the population, the incidence of proximal humeral fracture (PHF) has increased. However, the optimal method for open reduction and internal fixation (ORIF) remains controversial.

**Methods:**

We performed a retrospective analysis of patients with PHF who underwent locking plate internal fixation at our institution from January 2016 to December 2018. Patients were divided into two groups based on the surgical approach used: an expanded deltoid-split approach group (ORIF group) and minimally invasive deltoid-split approach group (minimally invasive percutaneous plate osteosynthesis, [MIPPO] group). The groups were compared in terms of demographic and perioperative characteristics, and clinical outcomes.

**Results:**

A total of 115 cases of PHF were included in our study, of which 64 cases were treated using the minimally invasive deltoid-split approach and 51 using the extended deltoid-split approach. Fluoroscopy was performed significantly less frequently in the ORIF group and the surgical duration was shorter. However, the postoperative visual analogue scale (VAS) pain score and duration of postoperative hospital stay were significantly higher compared to the MIPPO group. Moreover, secondary loss was significantly less extensive in the ORIF group compared to the MIPPO group, while there was no significant group difference in fracture healing time, Constant shoulder score, or complications at the last follow-up visit.

**Conclusions:**

The clinical outcomes associated with both the minimally invasive and extended deltoid-split approaches were satisfactory. The data presented here suggest that the extended deltoid-split approach was superior to the minimally invasive deltoid-split approach in terms of operational time, fluoroscopy, and secondary loss of reduction, while the minimally invasive approach was superior in terms of postoperative pain and hospital stay. Accordingly, neither procedure can be considered definitively superior; the optimal surgical procedure for PHF can only be determined after full consideration of the situation and requirements of the individual patient.

## Background

Proximal humeral fracture (PHF) is one of the most common type of fractures, accounting for 4–5% of all adult fractures [1, 2]. Furthermore, it is estimated that the number of PHF cases will triple in the next 10 years due to the rapid aging of population [3]. While the choice between operation or conservative treatment for PHF remains controversial, the development of of new internal fixation technologies has led to more PHF patients achieving a satisfactory functional outcome after the operation [4–6].

Open reduction and internal fixation (ORIF), minimal invasive percutaneous plate osteosynthesis (MIPPO), intramedullary nail internal fixation and arthroplasty are the most common surgical interventions for PHF [7], with ORIF with locking plate being the most common [8]. The conventional deltopectoral approach is gradually being replaced by deltoid-split approach, due to the extensive soft tissue and muscle dissection required to expose the lateral aspect of the humerus [9, 10]. In contrast, the deltoid-split approach utilizes the muscle space between the anterior and middle heads of the deltoid muscle, enabling sufficient exposure of both the greater and the lesser tuberosities [11]. The minimally invasive deltoid-split approach can alos further reduce soft tissue damage, allowing patients to exercise the affected shoulder joint earlier. However, recent studies have reported that the use of the MIPPO technique in combination with the deltoid-split approach may affect the blood supply to the humeral head and cause axillary nerve injury [12, 13].

Previous studies have compared the clinical results of ORIF with both the conventional deltopectoral and deltoid-split approach for PHF [14–16]; however, few studies have compared the effect of extended and minimally invasive deltoid-split approach on clinical outcomes. Therefore, we performed a retrospective analysis comparing clinical outcomes between surgical intervention types in PHF patients.

## Methods

This study was approved by our institutional ethics committee, with informed consent obtained from all participants prior to enrollment. The inclusion criteria were as follows: The inclusion criteria were as follows: closed PHF patients ≥18 years of age who underwent PHILOS plate fixation (Synthes, Oberdorf, Switzerland) at our institution, had complete electronic medical records, and had no history of injury to the ipsilateral upper extremity, and no evidence of multiple traumas or pathological fractures. From January 2016 to December 2018, 146 consecutive PHF patients were fixed with PHILOS plate at our institution. Of these patients, 31 were excluded due to open fractures (n = 1), history of injury to the ipsilateral upper extremity (n = 3), multiple traumas (n = 3), pathological fractures (n = 2), or loss to follow-up (n = 22).. Thus, the final cohort comprised 115 patients.

The operation was carried out in the beach chair position under general anesthesia (GA) or brachial plexus anesthesia (BA). The image intensifier was placed on the opposite side to meet the requirement for views of various positions during the operation. The surgical approach used for each patient was determined by the operating surgeon, with all operations performed by the same team (which has more than 15 years of experience in the treatment of PHF). For the minimally invasive deltoid-split approach, a ~ 4 cm longitudinal incision was made in the skin under the acromion along the anterolateral side, and separated in the direction of the deltoid muscle fiber to exposed the upper part of humeral head. The bone was then pushed along the humeral shaft to the distal end of the fracture to establish a soft tissue channel. During the operation, close attention was necessary to protect the axillary nerve and its accompanying blood vessels. In addition, a distal window ~ 3 cm in length was created to facilitate insertion of the distal screw, and expanded until the axillary nerve could be identified. For the extended deltoid-split approach, an ~ 8 cm longitudinal incision was made in the skin under the acromion along the anterolateral side, to expose the deltoid muscle. The muscle fibers were separated longitudinally along the anterior and the middle heads of the deltoid to expose the axillary nerve and its accompanying vessels, which were protected during the operation, with expansion continuing until the fracture was exposed. According to the standard method of fracture reduction, a PHILOS plate of appropriate length and matching screws were used for fixation. During the operation, photographs were taken from different angles to check for screw cut-out. In cases with rotator cuff, it was repaired during the operation.

All patients underwent similar postoperative rehabilitation: a forearm sling was used for 3–6 weeks to relieve the discomfort caused by postoperative limb hanging, and passive mobilization exercise (pendulum exercise, flexion, and external rotation) were performed for 1–6 weeks according to fracture type and bone healing status. Stretching and resistance training was recommended until the fracture had fully healed.

**Fig. 1:**
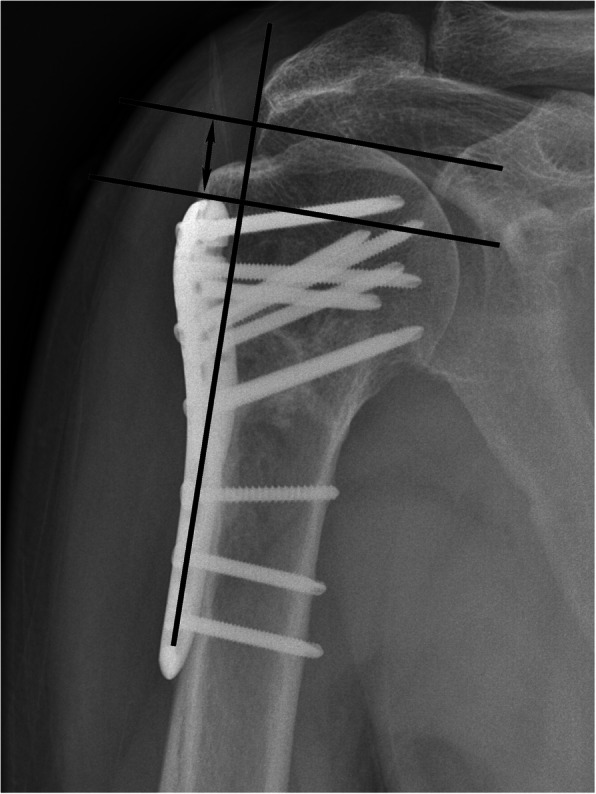
The method of measuring the distance between the humeral head and the proximal end of the plate. Legend: The distance between two straight lines orthogonal to the plate axis was measured, one straight line passed through the proximal end of the plate and the other through the top of the humeral head, as shown in the black double arrow

The following demographic data were collected: sex, age, height, weight, comorbidities (i.e., hypertension, diabetes, and cardiopathy), mechanism of injury (low-energy injuries [falls from standing height or less] and high-energy injuries [e.g., traffic accidents and falls from greater heights]), injured side (dominant or non-dominant side), time to surgery, Neer fracture classification [17], medial support status (complete or incomplete medial support), type of anesthesia (GA or BA), American Society of Anesthesiologists (ASA) scores, use of intraoperative fluoroscopy, surgical duration, visual analogue scale (VAS) pain score on the first day after surgery, postoperative hospital stay, fracture healing time, follow up duration, Constant shoulder score at the last follow-up visit, and secondary loss of reduction (determined based on anterior and posterior images of the shoulder joint obtained after the operation and at the last follow-up visit. The distance between two straight lines orthogonal to the plate axis was measured, with one straight line passed through the proximal end of the plate and the other through the top of the humeral head [18], Fig. 1). Complications including wound infection, delayed union, subacromial impingement syndrome, screw cutting out, and humeral head necrosis. Secondary loss of reduction was independently measured twice each by two radiology physicians who did not participate in this study, and the average of the four results used in the statistical analysis.

### Statistical analysis

Data analysis was performed using SPSS 22.0 (SPSS Inc., Chicago, IL, USA). Descriptive statistics are presented as the mean ± standard deviation or number of cases and percentages. For the normally distributed continuous variables, the data were analyzed using the independent sample t-test (Student’s t-test) or the Mann-Whitney U test. The chi-square test was used to analyze qualitative variables, with p-values < 0.05 considered statistically significance.

## Results

A total of 115 cases of PHF patients were included in our study, of which 64 (55.7%) were treated using the minimally invasive deltoid-split approach (MIPPO group), and 51 (44.3%) using the extended deltoid-split approach (ORIF group). The anthropometric and demographic data of the two groups are shown in Table 1. There was no statistical difference between the group in sex, age, height, weight, comorbid diseases, mechanism of injury, or injury side.

**Table 1:** The differences of patients’ demographic information between two groups

Characteristic	ORIF group	MIPPO group	p value
Number of patients	51	64	
Sex (male/female)	25/26	27/37	0.465
Age (year)	62.02 ± 10.65	62.09 ± 12.74	0.973
Height (m)	1.64 ± 0.09	1.63 ± 0.07	0.281
Weight (Kg)	64.57 ± 8.45	63.50 ± 10.41	0.554
Hypertension	18 (35.29%)	17 (26.56%)	0.312
Diabetes	8 (15.69%)	8 (12.50%)	0.624
Cardiopathy	3 (5.88%)	2 (3.12%)	0.471
Mechanism of injury			0.440
Low-engery	42 (82.35%)	56 (87.50%)	
High-engery	9 (17.65%)	8 (12.50%)	
Injury side			0.114
Dominant side	36 (70.59%)	36 (56.25%)	
Non-dominant side	15 (29.41%)	28 (43.75%)	

Data are mean standard deviation or number of cases and percentages; ORIF, Open reduction and internal fixation; Mippo, Minimally invasive percutaneous plate internal fixation

The perioperative data of the two groups are shown in Table 2. There were no significant differences in fracture classification, medial support status, ASA score, or type of anesthesia, however, the incidence rates of intraoperative fluoroscopy (4.37 ± 0.72 versus 7.27 ± 0.93, p < 0.001) and surgical duration (62.94 ± 10.18 min versus 82.25 ± 12.36 min, p < 0.001) were significantly lower, while the postoperative VAS score was significantly higher (6.33 ± 1.05 versus 4.78 ± 1.16, p < 0.001), and the postoperative hospital stay significantly longer (5.14 ± 1.58 days versus 3.81 ± 1.08 days, p < 0.001), in the ORIF group compared to the MIPPO group.

**Table 2:** Comparison of perioperative indicators between two groups

Characteristic	ORIF group	MIPPO group	p value
Fracture classification			0.829
II	16 (31.37%)	18 (28.12%)	
III	21 (41.18%)	30 (46.88%)	
IV	14 (27.45%)	16 (25.00%)	
Medial support status			0.816
Complete	29 (56.86%)	35 (54.69%)	
Incomplete	22 (43.14%)	29 (45.31%)	
ASA score			0.501
I	10 (19.61%)	16 (25.00%)	
II	37 (72.55%)	40 (62.50%)	
III	4 (7.84%)	8 (12.50%)	
Type of anesthesia			0.878
BA	31 (60.78%)	38 (59.38%)	
GA	20 (39.22%)	26 (40.62%)	
Fluoroscopy times	4.37 ± 0.72	7.27 ± 0.93	***< 0.001***
Surgical duration (minute)	62.94 ± 10.18	82.25 ± 12.36	***< 0.001***
VAS	6.33 ± 1.05	4.78 ± 1.16	***< 0.001***
Postoperative hospital stay (day)	5.14 ± 1.58	3.81 ± 1.08	***< 0.001***

*ASA* American Society of Anesthesiologists, *BA* Brachial plexus anesthesia, *GA* General anesthesia, *VAS* Visual Analogue Scale

Bold and bold font represent P < 0.05

**Fig. 2:**
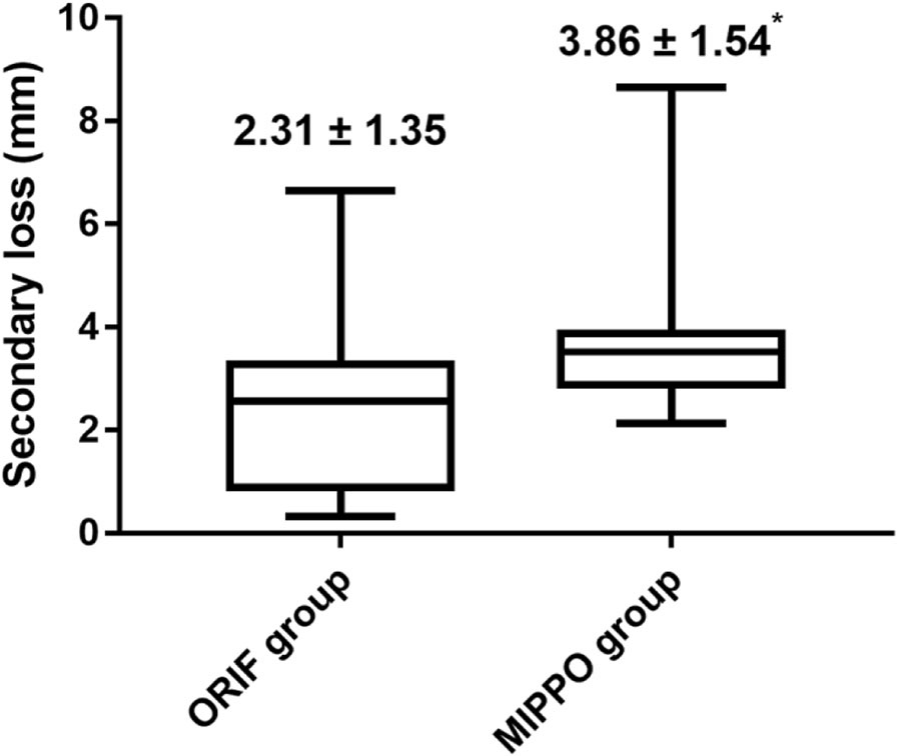
Comparison of secondary loss of reduction between two groups. Legend: ORIF, Open reduction and internal fixation; Mippo, Minimally invasive percutaneous plate internal fixation; ^*^ indicate p < 0.05

We followed patients in the ORIF group and MIPPO group for 16.04 ± 2.93 months and 16.25 ± 3.30 months, respectively. No significant differences in fracture healing time, shoulder function score, or clinical complications were evident between the groups at the time of the last follow-up (Table 3). In the MIPPO and ORIF group, there were one and two cases of superficial tissue infection, respectively, with all infection controlled by local dressing change and antibiotic injection. There were two and three cases of delayed union in the MIPPO and ORIF groups, respectively, although all fractures did heal eventually. Subacromial impingement syndrome was observed in one MIPPO and two ORIF patients, with symptoms improving after internal fixation was removed. In the MIPPO group, one patient experienced screw cut-out, which required reoperation to resolve. One case of humeral head necrosis was observed in the MIPPO group during long-term follow-up, which was treated via shoulder hemiarthroplasty (Table 3). In addition, secondary loss of reduction was significantly less extensive in the ORIF group compared to the MIPPO group (2.31 ± 1.35 versus 3.86 ± 1.54, p < 0.001, Fig. 2). Notably, none of the patients had neurologic complications.

**Table 3:** Comparison of follow-up information between two groups

Characteristic	ORIF group	MIPPO group	p value
Healing time (month)	3.69 ± 0.84	3.73 ± 1.10	0.797
Follow up time (month)	16.04 ± 2.93	16.25 ± 3.30	0.721
Functional score	86.49 ± 8.44	83.75 ± 10.38	0.130
Secondary loss (mm)	2.31 ± 1.35	3.86 ± 1.54	***< 0.001***
Complication			–
Wound infection	2	1	
Delayed union	3	2	
SIS	2	1	
Screw cutting out	0	1	
Head necrosis	0	1	

*SIS* Subacromial impingement syndrome

Bold and bold font represent P < 0.05

## Discussion

PHF is one of the most common fracture types in adults. Generally, the purpose of treatment for patients with surgical indications is to restore the stability of fracture and the function of the shoulder joint [19]. The conventional deltopectoral approach for PHF readily exposes the glenohumeral joint; however, access to the fracture location is limited. Moreover, the operation often requires extensive soft tissue peeling, which significantly influences the blood supply of the fracture site and can easily exacerbate ischemic necrosis of the humeral head. Moreover, detachment of the front edge of the deltoid muscle causes postoperative shoulder pain and affects the ability of patients to perform early functional exercises [14, 20, 21]. With the deltoid-split approach, there is no need to cut the deltoid fiber, so there will be no affect on shoulder flexion or abduction. This approach is therefore convenient for exposure of both the greater and the lesser tuberosities, and it is conducive to the reduction of fractures [11]. The minimally invasive deltoid-split approach can further reduce the damage to soft tissue, allowing patients to exercise the affected shoulder joint earlier compared to other procedures [15]. However, the minimally invasive deltoid-spilt approach have some disadvantages, including a risk of axillary nerve injury, and indirect reduction will increase the radiation duration during the operation [3] To the best of our knowledge, no previous study has compared the clinical results of the extended deltoid-split and minimally invasive deltoid-split approaches in the treatment of PHF; such a study is therefore necessary.

The frequency with which intraoperative fluoroscopy was performed was significantly lower, while the surgical duration was significantly shorter, in the ORIF group compared to the MIPPO group, consistent with previous reports [22]. We believe that the minimally invasive approach used in MIPPO indirectly reduces the fracture around the incision, which may extend the surgical duration and the increase the requirement for intraoperative fluoroscopy. Moreover, postoperative VAS score and duration of postoperative hospital stay were both significantly higher in the ORIF group relative to the MIPPO group. These results were likely due to the minimally invasive deltoid-split approach requiring less soft tissue peeling compared to the extended deltoid-split approach, resulting in a corresponding reduction in postoperative pain and hospital stay length. No significant group difference in shoulder function score was evident at the time of the last follow-up, with most patients reporting satisfactory functional recovery. Therefore, we believe that there was no significant difference between the two surgical approaches in terms of treatment outcomes, with both methods proving effective.

According to previous reports, the total incidence of complications after internal fixation of PHF is 10–34% [23–25]. In our study, the total incidence of complications in the two groups was 13.7 and 9.4% respectively. Buecking et al. [14] previously reported that the incidence of postoperative complications of PHF was highly correlated with surgeon experience. However, it is worth mentioning that we observed significantly less extensive secondary loss of reduction in the ORIF group compared to the MIPPO group (2.31 ± 1.35 mm versus 3.86 ± 1.54 mm, p < 0.001). Gardner et al. [26, 27] suggested that the secondary loss of reduction associated with PHF was in turn related to the integrity of the medial support, where incomplete medial support might increase the loss of reduction. Osterhoff et al. [18] analyzed the clinical data of 44 patients with PHF treated by internal fixation via the minimally invasive deltoid-split approach, and founded that the reduction loss (0.77 ± 1.44 mm) of the patients with a calcar screw was significantly lower than that of those without inserted calcar screw (2.56 ± 2.65 mm; P = 0.01). One possible explanation for this result may be the difficulty of inserting the calcar screw under the minimally invasive deltoid-split approach. In patients with incomplete preoperative medial support, the likelihood of insufficient calcar screw support during the operation may be higher, leading to an increased risk of postoperative reduction. Moreover, to avoid damage to the axillary nerve, some surgeons tend to avoid the placement of the calcar screw, especially when using the minimally invasive percutaneous plating [28]. The risk of axillary nerve injury during minimally invasive surgery is a significant concern in MIPPO. Acklin et al. reported a significant risk of axillary nerve injury when using MIPPO, whereas Koljonen et al. was able to apply MIPPO without axillary nerve injury [3]. To protect the axillary nerve in the subdeltoid bursa, Buecking et al. [14] used the index finger to trace the course on the skin surface. Ruchholtz et al. [29] placed the distal screw in the distal three holes of the plate, to keep the screws as far away from the axillary nerve as possible. No patients with axillary nerve injury were identified during follow-up, which supports the precautions taken to protect the axillary nerve during the operation, whether ORIF was used to expose the axillary nerve, or MIPPO to reduce the fracture indirectly.

Our study has several limitations. First, it used a retrospective design and the sample size was relatively small. Thus, a prospective study examining a larger cohort of patients will be necessary to verify the results. Second, the surgical approach for each patient was determined by the surgeon, which may have led to bias. Third, we only recorded VAS pain scores on the first day after surgery, thereby neglecting the minimal clinically important difference of pain in PHF. Finally, some patients showed poor compliance and a lack of appropriate rehabilitation, which may have affected the postoperative functional recovery.

## Conclusions

The clinical results of minimally invasive and extended deltoid-split approaches were both satisfactory. The minimally invasive deltoid-split approach is superior in terms of postoperative pain and hospital stay, while with the extended deltoid-split approach the surgical duration is shorter and the requirement for fluoroscopy is lower. Notably, our preliminary data showed that patients suffered more extensive secondary loss of reduction in the context of the minimally invasive deltoid-split versus extended deltoid-split approach, although further studies will be needed to verify these results. Based on the data presented here, neither procedure can be considered definitively superior; the optimal surgical procedure for PHF can only be determined after full consideration of the situation and requirements of the individual patient.

## Data Availability

The datasets analyzed in the study are available from the corresponding author on reasonable request.

## Abbreviations

PHF: Proximal humeral fracture
ORIF: Open reduction and internal fixation
MIPPO: Minimally invasive percutaneous plate internal fixation
VAS: Visual Analogue Scale
GA: General anesthesia
BA: Brachial plexus anesthesia
ASA: American Society of Anesthesiologists
